# The Interdialytic Creatinine Rise is a novel marker of volume overload and mortality risk in hemodialysis patients

**DOI:** 10.1186/s12882-018-1008-0

**Published:** 2018-08-16

**Authors:** Ljubomir M. Ilic, Roger B. Davis, Robert S. Brown, Stewart H. Lecker

**Affiliations:** 1 Nephrology Division, Department of Medicine, Beth Israel Deaconess Medical Center, Boston, MA USA; 2 Division of General Medicine and Primary Care, Beth Israel Deaconess Medical Center and Harvard Medical School, Boston, MA USA; 3 Department of Medicine - Division of Nephrology and Hypertension, University of Florida College of Medicine – Jacksonville, 655 West 8th Street, Jacksonville, Florida USA

**Keywords:** Dialysis volume, Hemodialysis, End stage kidney disease, Volume overload, Dialysis mortality, Risk indicator

## Abstract

**Background:**

Volume overload poses a major risk in hemodialysis patients but simple detection methods are lacking. We propose a novel marker, the Interdialytic Creatinine Rise (IDCR), readily calculated as the change in serum creatinine over time (in mg/dL/h), to assess volume overload and predict mortality risk in hemodialysis patients.

**Methods:**

First, we calculated IDCR changes with volume in a prospective cohort of 35 hospitalized hemodialysis patients awaiting hemodialysis and 33 hospitalized patients undergoing hemodialysis every other day. Second, in a prospective cohort of 25 outpatients, IDCR cutoff values associated with hypervolemia were determined between two treatments and compared with simultaneous volume assessments by their nephrologist. Third, IDCR as a mortality predictor was studied using survival analysis in a longitudinal retrospective cohort study of 39 maintenance hemodialysis patients followed from 2012 until death or 2017.

**Results:**

IDCR decreased by − 0.014 mg/dL/h each day (95%CI − 0.017,− 0.010; *p* < 0.001) without dialysis due to fluid volume gain and increased by 0.013 mg/dL/h (95%CI 0.008,0.017; *p* < 0.001) from before to after each successive hemodialysis due to fluid removal.

Choosing an IDCR cutoff value of ≤0.1 had sensitivity of 82% and specificity of 79% in diagnosing volume overload with the area under the ROC curve of 0.78 (95%CI 0.59,0.97).

The hazard ratio of death for each 0.01 decrease in IDCR was 1.64 (95%CI 1.31,2.07; *p* < 0.001). If IDCR decreased to less than 0.05 mg/dL/h, the median survival was 32 days and the odds ratio of death within 2 months was 38 (95%CI 8, 131; *p* < 0.001).

**Conclusions:**

In this pilot study, IDCR is shown to be a novel metric that decreases with fluid retention and increases after fluid removal. IDCR can assist clinicians in detection or exclusion of volume overload in hemodialysis patients and provide prognostic value in identifying those at high risk for death.

**Electronic supplementary material:**

The online version of this article (10.1186/s12882-018-1008-0) contains supplementary material, which is available to authorized users.

## Background

Volume overload is a well-established cardiovascular risk factor in chronic hemodialysis patients and yet can be difficult to identify clinically at the point-of-care [1, 2]. Different markers such as interdialytic weight gain, BNP, relative plasma volume monitoring, and bioimpedance spectroscopy have been used to assess volume in this patient population [3–5]. Each of the methods has its own shortcomings and has not proved to reduce the excess risk and mortality in the few existing goal-oriented clinical trials. Two studies published this year utilized bioimpedance spectroscopy to evaluate volume overload and demonstrate its association with mortality in chronic dialysis patients [6, 7]. However, bioimpedance devices have not been generally accepted by the major dialysis providers and no device is approved for use in the United States. We hypothesized that a novel measure to detect volume overload, the Interdialytic Creatinine Rise or IDCR, could be simply calculated from routine and inexpensive laboratory values. After a hemodialysis session, creatinine concentration reaches a nadir following equilibration between blood, extravascular and tissue fluid stores. Creatinine then starts to increase due to new generation and minimal renal clearance, reaching its peak before the next hemodialysis session. The rate of increase of serum creatinine concentration between hemodialysis sessions depends upon two opposing processes – net creatinine retention due to its production with minimal excretion opposed by fluid intake increasing the total body volume diluting the creatinine concentration. As creatinine production remains relatively constant in any given patient, the rate of serum creatinine rise is largely related to accumulated volume. Therefore, we theorized that this new measure, the IDCR, calculated as the difference between two serum creatinine concentrations from the same interdialytic period divided by the time difference of respective blood sample collections (expressed in mg/dL per hour), would be closely related to total body volume and could serve as a marker of volume overload. In this pilot study, we have demonstrated that low IDCR values are associated with volume overload, and when very low, are also indicative of a markedly increased risk of patient mortality.

## Methods

**Fig. 1:**
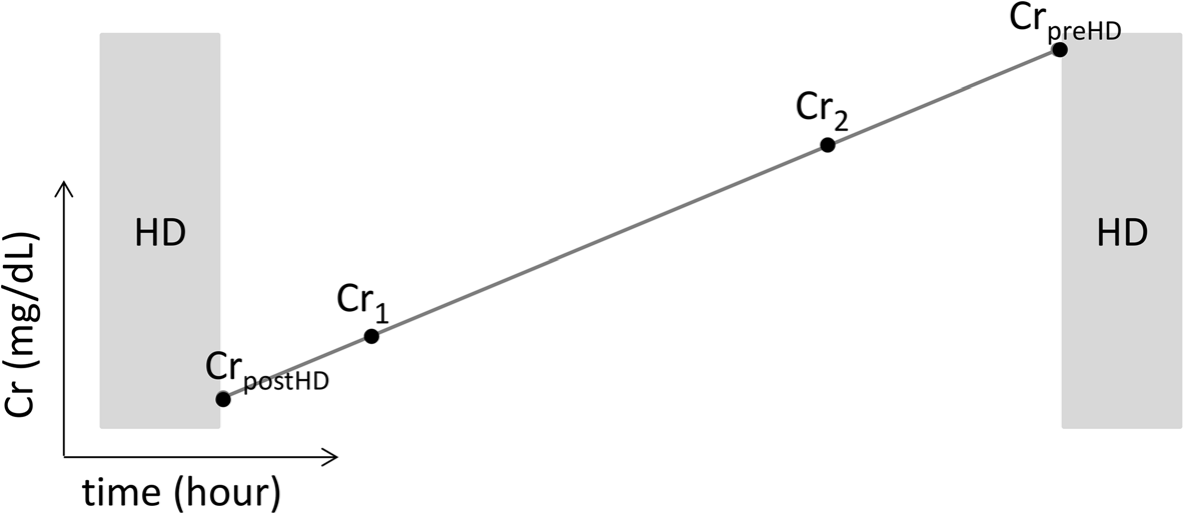
Conceptual calculation of IDCR using interdialytic serum creatinine values (Cr) obtained at recorded times (t). If the immediate post- and pre- dialysis creatinine values are obtained, IDCR is calculated as IDCR = (Cr_preHD_ – Cr_postHD_) / (t_preHD_ – t_postHD_). Otherwise IDCR is calculated from the creatinine values in the same interdialytic period as, IDCR = (Cr_2_ – Cr_1_) / (t_2_ – t_1_)

The rate of creatinine rise or IDCR (mg/dL/h) was calculated as the difference between two serum creatinine concentrations obtained at least 18 h apart within the same interdialytic period divided by the time between the two blood draws (see Fig. 1). IDCR was compared between successive hemodialysis treatments to show the effect of volume removal by ultrafiltration. IDCR was recalculated daily from the daily creatinine values if hemodialysis was delayed to show the effect of volume gain. The scientific foundation of IDCR is presented in the Supplement along with simulations of interdialytic volume gain (Additional file 1) [8–12].

### Study population and design

#### Study 1: Evaluation of IDCR changes with volume in hospitalized hemodialysis patients

We followed 47 patients recruited after admission to the Beth Israel Deaconess Medical Center in April 2016 during their hospital stay. Patients were included only if they had been on maintenance hemodialysis for at least one year, reported minimal urine output, did not undergo renal transplantation or major amputations, and who were followed at our institution. Our hospital goal was to provide hemodialysis every other day three times per week. Most of the patients waited 1, 2 or 3 days for hemodialysis, but there were four who waited longer due to malfunctioning vascular accesses. Overall, it took 1 to 5 days to provide hemodialysis in 35 of the 47 patients, comprising group 1. There were 33 patients in group 2 followed while undergoing up to three consecutive hemodialysis sessions over a week (of which 21 patients overlapped between the two groups). Their demographics, clinical characteristics, and creatinine values with times of blood draws were collected using chart review. For each patient, the IDCR at different times was calculated as IDCR = (Cr_2_ – Cr_1_) / (t_2_ – t_1_) using daily creatinine values obtained routinely during the hospital admission. The linear regression models were fit using generalized estimating equations (GEE) methods to account for the longitudinal repeated measures design and to study IDCR changes over time in the presence or absence of hemodialysis treatments.

#### Study 2: Evaluation of IDCR as a marker of volume overload in maintenance hemodialysis outpatients

After analyzing the cohort described above, we recruited 25 patients at their outpatient dialysis unit (DaVita, Brookline, MA) in March of 2017 and followed them prospectively over two consecutive dialysis sessions, either Monday to Wednesday, or Tuesday to Thursday. Each patient consented to two blood draws from which IDCR was calculated as (Cr_preHD_ – Cr_postHD_) / (t_preHD_ – t_postHD_). Cr_postHD_ was measured in the blood sample obtained immediately after dialysis following the standard dialysis unit protocol for measurement of post BUN. Cr_preHD_ was measured in the blood sample obtained immediately before the following dialysis session. We collected patient data including demographics, clinical characteristics, and creatinine values with collection times using chart review. Volume of each patient as overloaded or not was assessed by his/her long-term nephrologist. The clinician’s assessment of volume status was based upon one or more findings of increased shortness of breath over the baseline, lower extremity edema, signs of pulmonary fluid presence, or weights significantly over the suspected “dry weight”. The assessment of volume was confirmed over at least three serial time-points up to and including the week of the blood draw to maximize intra-rater reliability. We calculated the sensitivity and specificity of different IDCR cutoffs to detect volume overload using the nephrologist’s volume assessment as the gold standard. Clopper-Pearson method was used to construct binomial confidence intervals for the various proportions calculated including the sensitivity and specificity, and for the area under the ROC curve [13].

#### Study 3: Evaluation of IDCR as a predictor of mortality

We identified a retrospective cohort of 39 maintenance hemodialysis patients that had been admitted to our hospital in 2012 and were followed longitudinally during their readmissions to our hospital until their death or censorship in March of 2017. Using chart review, we collected their data including demographics, clinical characteristics, date of death and creatinine values with times drawn on the first two days of their admissions to calculate serial IDCR values. The data were analyzed using Cox proportional hazards model treating IDCR as a time varying covariate. In the same study, the odds ratio was estimated from the logistic regression model fit using generalized estimating equations to account for the longitudinal repeated measures design.

### Statistical analyses

The statistical analyses were performed using SAS statistical software (version 9.4 for Windows, SAS institute, Inc., Cary, NC). The plots were created with Stata (version IC 14 for Windows, StataCorp LLC, College Station, Texas).

The studies were approved by the Committee on Clinical Investigations at Beth Israel Deaconess Medical Center in Boston, MA and completed in accordance to the Institutional review board (IRB) Protocol number: 2016P000052. DaVita facility in Brookline, MA ceded their review to the IRB at BIDMC.

## Results

### Study 1: Evaluation of IDCR changes with volume in hospitalized hemodialysis patients

**Fig. 2:**
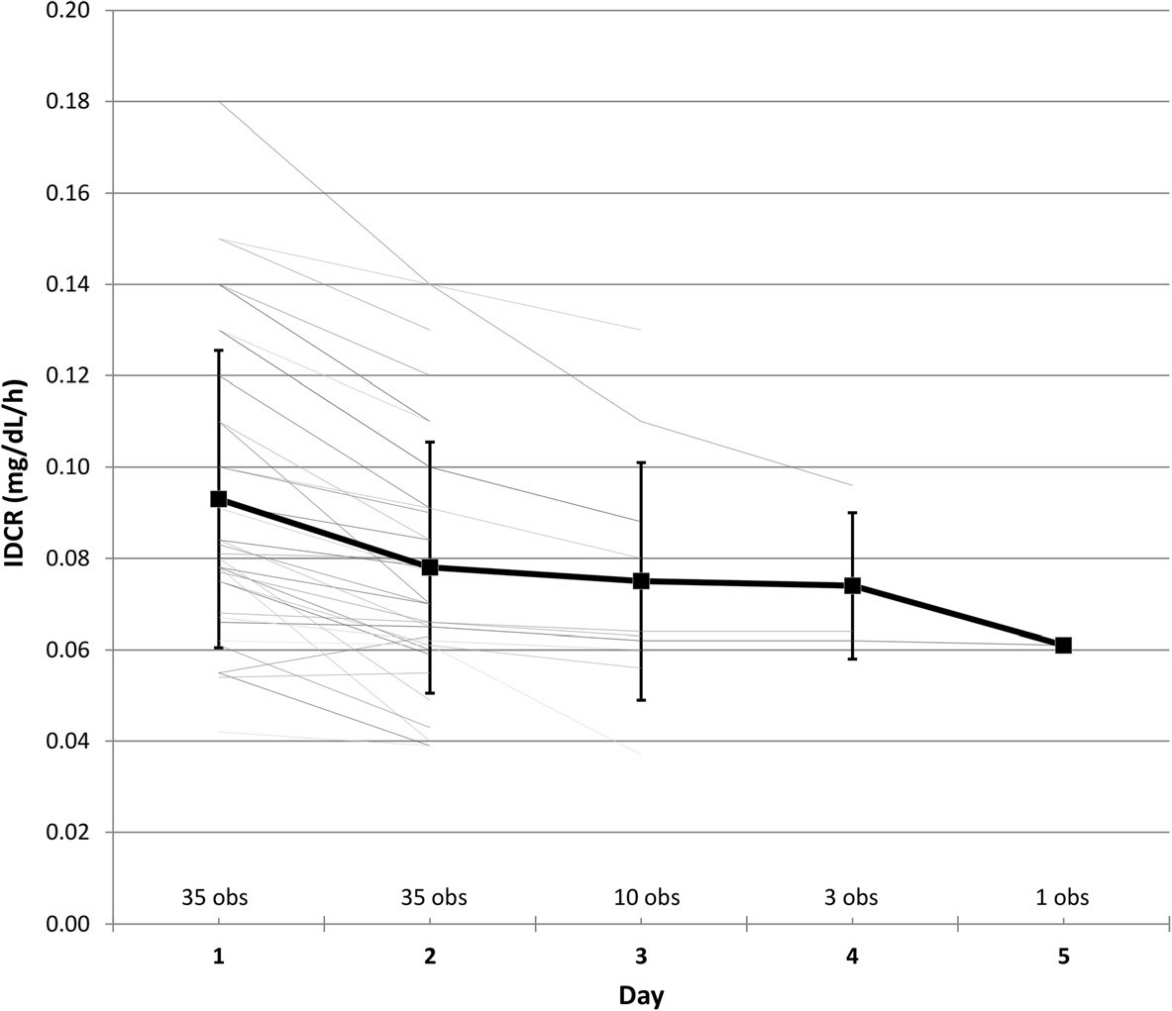
IDCR change with time in 35 hospitalized patients without hemodialysis (Group 1 of Study 1). IDCR is calculated on hospital day 1 and each following day awaiting hemodialysis. The thin lines identify individual patients, with the number of observations (obs total of 84) for the day shown below. The average values of IDCR are shown by the bold squares with the error bars corresponding to the standard deviations. Based on the GEE model, IDCR decreases, changing by − 0.014 per day (95% CI − 0.017, − 0.010; *p* < 0.001) without HD due to volume gain

We tested the hypothesis that the calculated IDCR decreases as volume is gained in patients awaiting hemodialysis and increases after hemodialysis as volume is removed by ultrafiltration. The patients’ characteristics and IDCR values of the first group of 35 hospitalized patients followed serially while awaiting hemodialysis for up to five days are presented in Table 1. Based on the linear regression model fit using generalized estimating equations, IDCR decreases, changing by − 0.014 mg/dL/h per day without HD (95% CI − 0.017, − 0.010; *p* < 0.001) due to volume gain (Fig. 2). Age, gender, race and time on hemodialysis were considered as potential covariates and were not found to contribute to the estimate.

**Table 1:** Characteristics of inpatients followed longitudinally for up to five days while awaiting hemodialysis (Group 1 of Study 1)

Number of patients, n	35^b^
Age, mean ± SD, years	55 ± 14
Women, n (%)	16 (45)
Race, n (%)	
White	24 (68)
Black	6 (17)
Hispanic	4 (12)
Asian	1 (3)
Time on hemodialysis, mean ± SD, years	4.5 ± 1.9
Mean IDCR ± SD, mg/dL/h	
Hospital day 1 (35 obs)	0.093 ± 0.033
Hospital day 2 (35 obs)	0.078 ± 0.028
Hospital day 3 (10 obs)	0.075 ± 0.026
Hospital day 4 (3 obs)	0.074 ± 0.016
Hospital day 5 (1 obs)	0.061 ±^a^

Number of longitudinal observations (obs) is shown at each hospital day

^a^unable to calculate SD as one observation only

^b^cohort presented here has 21 patients in common with the cohort presented in Table 2

**Fig. 3:**
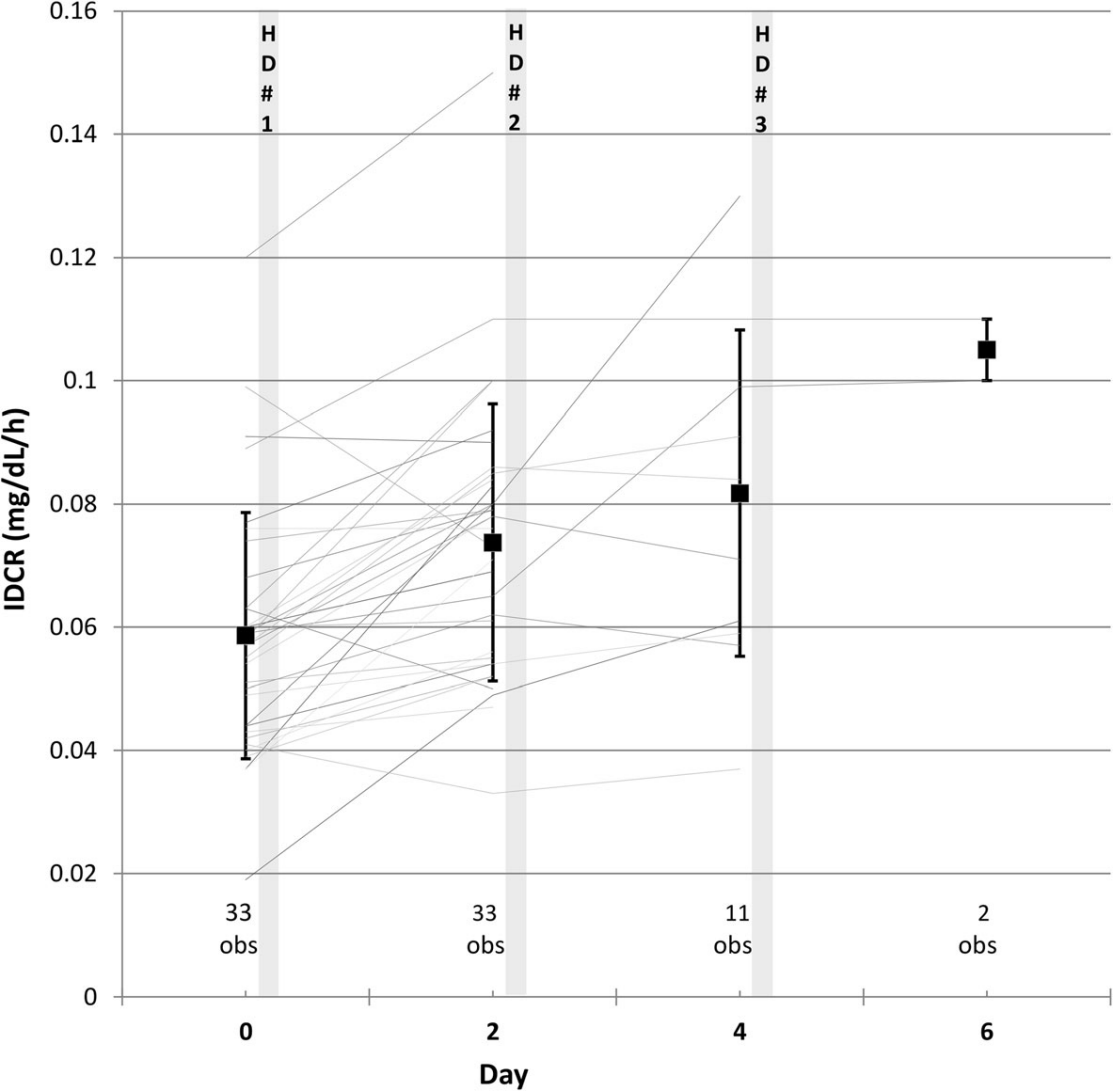
IDCR changes in 33 hospitalized patients after 1 to 3 successive hemodialysis treatments (Group 2 of Study 1). IDCR is calculated at baseline before the first hemodialysis session (Day 0), on Day 2 before the second hemodialysis session, on Day 4 before the third hemodialysis session, and on Day 6 following the third hemodialysis session. The thin lines identify individual patients and the number of observations (obs total of 79) for the day are shown below. The average values of IDCR are shown by the bold squares with the error bars corresponding to the standard deviations. Based on the GEE model, IDCR increases by 0.013 (95% CI 0.008, 0.017; *p* < 0.001) between each successive hemodialysis session due to fluid removal by ultrafiltration

The patients’ characteristics and IDCR values of the second group of 33 hospitalized patients followed serially for up to one week while undergoing hemodialysis sessions every other day are presented in Table 2. IDCR increases from before to after each successive hemodialysis session by a mean of 0.013 mg/dl/h (95% CI 0.008, 0.017; *p* < 0.001) due to fluid removal by ultrafiltration (Fig. 3). As the average volume of ultrafiltration was 2.5 ± 0.4 L per hemodialysis session, IDCR increases by an average of 0.013 mg/dL/h per 2.5 L removed or 0.005 (95% CI 0.003, 0.007) mg/dL/h per liter of fluid removed. Age, gender, race and time on hemodialysis were considered as potential covariates and were not found to contribute to the estimate.

**Table 2:** Characteristics of inpatients followed longitudinally for up to one week while undergoing hemodialysis every other day (Group 2 of Study 1)

Number of patients, n	33^b^
Age, mean ± SD, years	58 ± 15
Women, n (%)	14 (41)
Race, n (%)	
White	21 (64)
Black	8 (24)
Hispanic	3 (9)
Asian	1 (3)
Time on hemodialysis, mean ± SD, years	4.2 ± 2.1
Mean IDCR ± SD, mg/dL/h	
Baseline (33 obs)^a^	0.059 ± 0.020
One HD session (33 obs)	0.074 ± 0.023
Two HD sessions (11 obs)	0.082 ± 0.027
Three HD sessions (2 obs)	0.105 ± 0.005

Number of longitudinal observations (obs) is shown at baseline and after each hemodialysis session

^a^calculated before first hemodialysis session after admission

^b^cohort presented here has 21 patients in common with the cohort presented in Table 1

### Study 2: Evaluation of IDCR as a marker of volume overload in maintenance hemodialysis outpatients

**Fig. 4:**
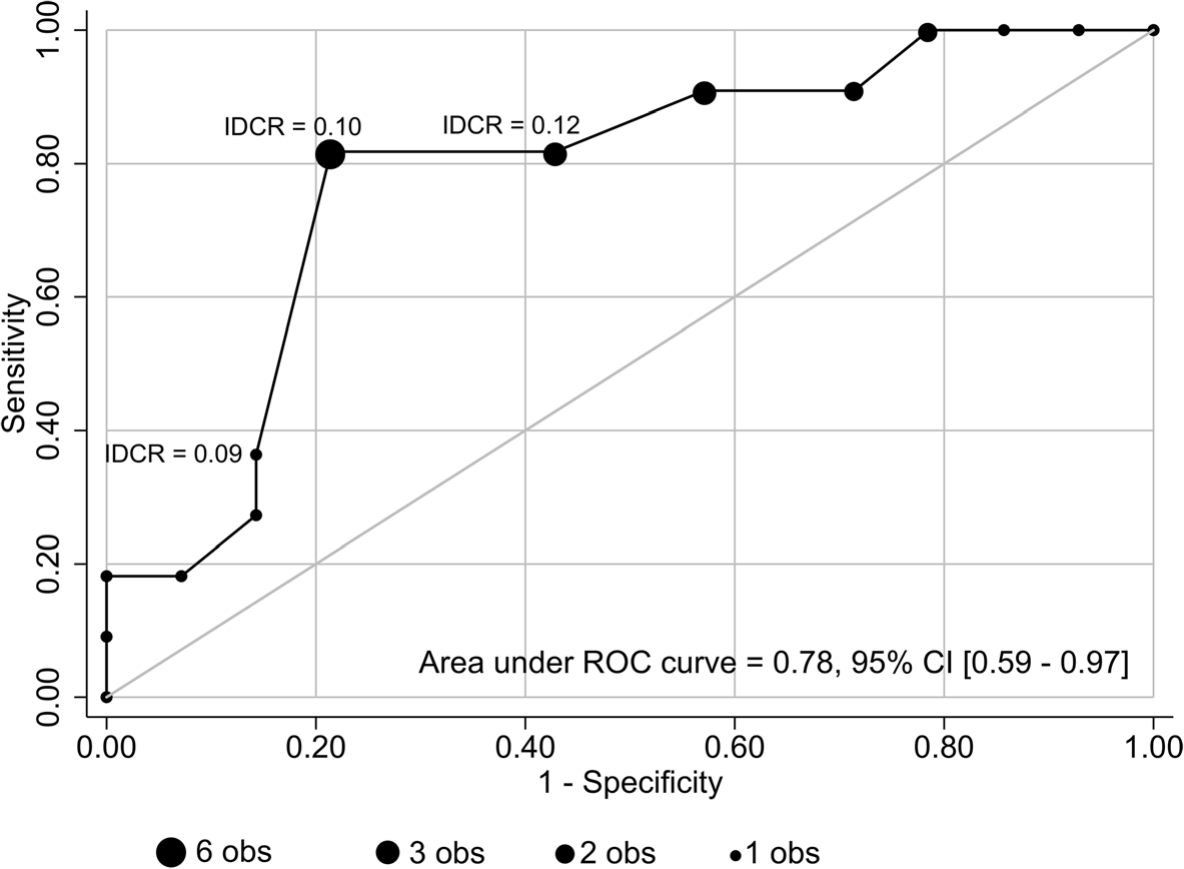
Receiver operating characteristic curve shows the sensitivity and specificity of IDCR measurements in 25 hemodialysis outpatients in Study 2 associated with or without volume overload as assessed clinically by their long-term nephrologist as the gold standard. The number of observations (obs) of each IDCR value of the 25 total observations is as shown. Area under the curve is shown with its 95% confidence interval

To be clinically useful, we tested the hypothesis that IDCR values associated with “acceptable” volume gains for hemodialysis patients could be used to detect and exclude “excessive” volume overload with high sensitivity and specificity. In the prospective cohort of 25 patients followed at their outpatient dialysis unit (Table 3), each had IDCR calculated during the first inter-dialytic period of the week. Different IDCR cutoff values were compared against the volume status of these 25 patients who were classified as either euvolemic or hypervolemic by their nephrologist. Since lower IDCR values are indicative of increasing total body volume, the test would be considered positive for volume overload if the measured IDCR was less than the specified IDCR cutoff value. The results for various IDCR cutoff values are shown on the receiver operating characteristic (ROC) curve in Fig. 4. The IDCR cutoff value providing the highest accuracy was determined to be 0.1 mg/dL/h; using an IDCR of ≤0.1 mg/dL/h to predict volume overload had a sensitivity of 82%, specificity of 79%, negative predictive value (NPV) of 85%, and positive predictive value (PPV) of 75%. With an accuracy of80%, the diagnostic ability of an IDCR of ≤0.1 mg/dL/h would alert the clinician to probable volume overload with the area under the ROC curve = 0.78 (95% CI 0.59, 0.97).

**Table 3:** Characteristics of maintenance hemodialysis patients in Study 2 studied to assess IDCR levels associated with or without volume overload

Number of patients, n	25
Age, mean ± SD, years	52 ± 18
Women, n (%)	12 (48)
Race, n (%)	
White	11 (44)
Black	7 (28)
Hispanic	5 (20)
Asian	2 (8)
Time on hemodialysis, mean ± SD, years	3.8 ± 2.4
Number with volume overload by nephrologist’s assessment (%)	11 (44)

### Study 3: Evaluation of IDCR as a predictor of mortality

**Fig. 5:**
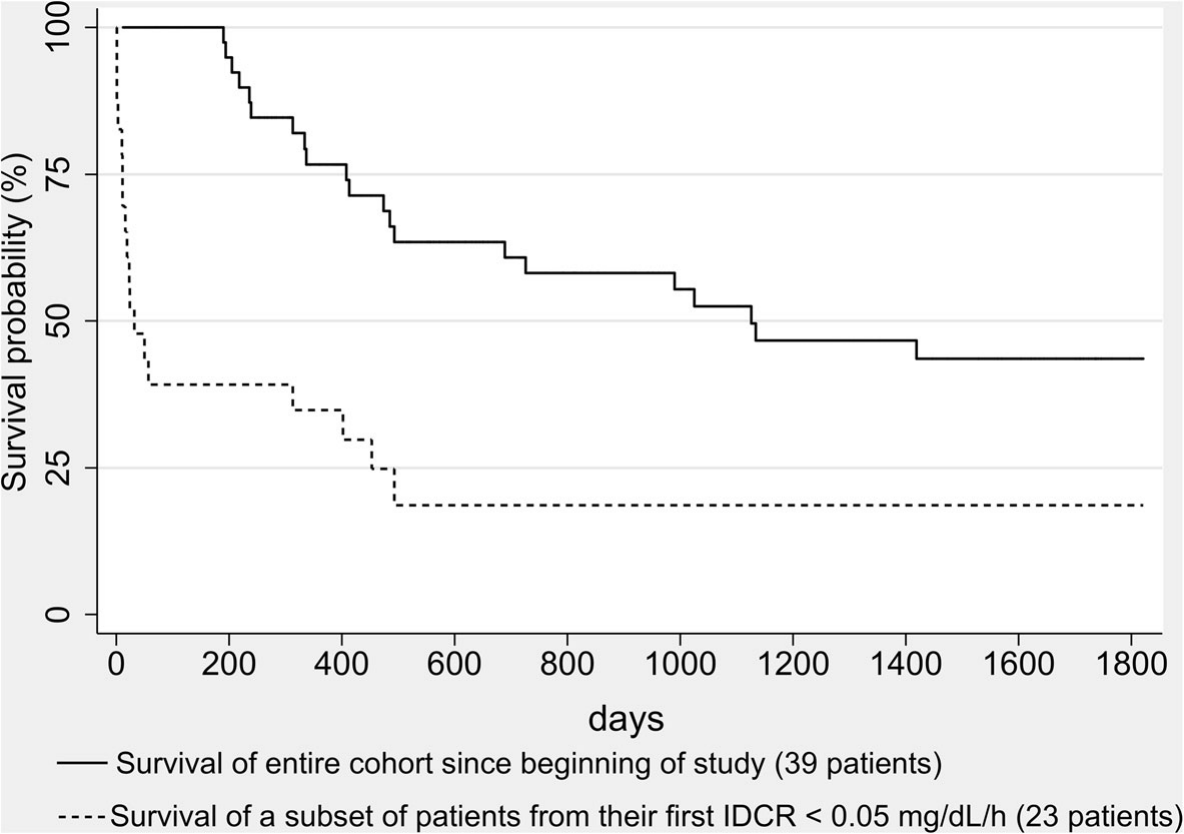
Kaplan-Meier survival curve of 39 hemodialysis patients in Study 3 followed longitudinally from 2012 until death or censorship in March of 2017 (solid line, number of subjects 39, 21 deaths), and of the subset of patients followed since their IDCR was less than 0.05 mg/dL/h (dashed line, number of subjects 23, 18 deaths)

Since excessive volume overload has been associated with an increased risk of death, we tested whether IDCR would be useful to identify patients who are at high risk. In the retrospective cohort of 39 patients followed from a hospitalization in 2012 until death or censorship in March of 2017, IDCR was calculated from routine creatinine values obtained on the first and second days of each hospital admission. The patients’ characteristics, clinical data and results are presented in Table 4. Fig. 5 shows a Kaplan-Meier survival curve of the entire cohort followed since the beginning of the study and the cohort’s subset of 23 patients followed since their first IDCR became less than 0.05 mg/dL/h. Using Cox proportional hazards regression model with IDCR as a time varying covariate, the hazard ratio of dying for each 0.01 mg/dL/h decrease in IDCR is 1.64 (*p* < 0.001; 95% CI 1.31, 2.07). Age, gender, race and time on hemodialysis, presence of systolic dysfunction (LVEF < 40%), and presence of coronary artery disease or peripheral artery disease were considered as potential covariates and were not found to contribute to the estimate. We then analyzed the patients whose IDCR fell below 0.05 mg/dL/h at any point in time during follow-up. The median survival after a patient’s IDCR decreased to below 0.05 mg/dL/h was 32 days. Based on the logistic regression model fit using generalized estimating equations, the odds ratio of dying within two months if IDCR decreased to below 0.05 mg/dL/h was 38 (*p* < 0.001; 95% CI 8, 131). The aforementioned covariates were also considered and did not influence these estimates.

**Table 4:** Characteristics of retrospective longitudinal study of Study 3 followed from a hospital admission in 2012 until death or censorship in 2017, stratified by IDCR < 0.05 at any time during follow up

	Total	IDCR < 0.05	IDCR > 0.05
Number of patients, n	39	23	16
Age, mean ± SD, years	66 ± 14	68 ± 13	64 ± 16
Women, n (%)	22 (56)	12 (52)	10 (62)
Race, n (%)			
White	21 (54)	13 (57)	8 (50)
Black	9 (23)	6 (26)	3 (19)
Hispanic	6 (15)	3 (13)	3 (19)
Asian	3 (8)	1 (4)	2 (12)
Years on hemodialysis at start of follow-up, mean ± SD	1.8 ± 0.5	1.9 ± 0.6	1.6 ± 0.5
Presence of CAD or PAD^c^, n (%)	20 (51)	9 (39)	11 (69)
LVEF^c^ < 40%, n (%)	6 (15)	2 (9)	4 (25)
Number of deaths during follow-up, n (%)	21 (54)	18 (78)	3 (19)
Median survival, days	1126	32^b^	Not calculated^a^

^a^unable to calculate median survival as there is no starting point in this cohort

^b^based on survival analysis from their first IDCR < 0.05

^c^*CAD* coronary artery disease, *PAD* peripheral artery disease, *LVEF* left ventricular ejection fraction

## Discussion

Evaluation of volume status in maintenance hemodialysis patients remains difficult and is often not called to the attention of the clinician until symptoms develop. Given the pervasiveness of the problem with few practical solutions in place, we have proposed a new simple measure of volume status in this patient population, the Interdialytic Creatinine Rise (IDCR), requiring only the measurement of two serum creatinine levels from a single interdialytic period. In this pilot study, we have shown that the IDCR decreases with each day awaiting hemodialysis due to fluid volume gain outpacing creatinine generation and retention. As expected, IDCR increases with each successive hemodialysis due to volume removal with ultrafiltration. As a clinically useful tool, an IDCR greater than 0.1 mg/dL/hr. has an excellent negative predictive value of 85% to exclude volume overload, while IDCR equal to or less than 0.1 mg/dL/h has a good positive predictive value of 75% to detect volume overload. In survival analysis, every decrement in IDCR of 0.01 mg/dL/h is associated with an increase in the hazard ratio of death by 1.64. If IDCR decreases to below 0.05 mg/dL/h, the odds ratio of dying in two months is 38 and the median survival in our study was only 32 days. While we believe that excessive volume retention is the major factor driving low IDCR values, we do not exclude, and presume that there may be, a contribution of decreased creatinine production in certain high-risk patients [14]. Either way, our study data have confirmed our mathematically-derived hypotheses that IDCR varies with body fluid volume, can help exclude or indicate possible volume overload, and has excellent prognostic value in identifying hemodialysis patients at high risk of death.

Of the existing markers of volume overload, interdialytic weight gain (IDWG) is commonly reported in outpatient dialysis units as a crude surrogate for volume overload and yet is a poor indicator of risk unless extreme [4]. An important distinction to make is that IDCR is not just a surrogate for IDWG or percentage weight gain from volume retention between dialysis treatments. Because the calculation of IDCR will mathematically yield a lower value if unrecognized fluid volume retention was present after a hemodialysis, even with the same interdialytic weight gain, the IDCR will provide a better indicator to alert the nephrologist to potential volume overload. Moreover, since IDCR is related to creatinine production as well as volume retention, it will be higher in healthier (and often heavier) patients with larger muscle mass and lower mortality risk even with larger interdialytic weight gains compared to patients with low IDCRs whether due to low muscle mass or volume overload who seem to be at higher risk for death. Despite the suggested utility of bioimpedance plethysmography and relative plasma volume monitoring, neither test is commonly used in practice as they are expensive and cumbersome while not adding significantly to the clinical assessment of volume alone. Measurements of the inferior vena cava diameter and plasma natriuretic peptides have limited clinical utility in this patient setting. In comparison, IDCR as a marker of volume is inexpensive, readily available, and poses no additional risk to the patient, as blood for creatinine measurements can be collected after and before dialysis from the existing circuit.

Major limitations of our pilot study include the relatively small sizes of our cohorts and limited number of potential confounders which may limit its generalizability. Several dialysis comorbidities, such as diabetes, COPD, and low albumin, were not considered as comparative mortality predictors. However, the sheer strength of association of IDCR with mortality is exceedingly high as noted above and likely greater than many other established mortality predictors. Other limitations include accuracy of the clinician assessment of volume overload as the “gold” standard, and perhaps, lower IDCR values leading to decreased sensitivity to indicate volume overload in hemodialysis patients with high residual renal function. Nevertheless, all three of our studies of IDCR were highly statistically significant, which speaks to the robustness of this new measure. It is possible that future larger studies may refine the IDCR cutoffs associated with hypervolemia and death. Also, IDCR could be compared to other volume measurements, such as bioimpedance plethysmography, when it becomes readily available, for further confirmation. However, we propose that our results are applicable to the majority of maintenance hemodialysis patients in chronic hemodialysis units, especially those with minimal residual kidney function. On the other hand, our results may be less likely to be applicable in intensive care unit settings or to cases of acute kidney injury in which creatinine production is varying.

## Conclusions

We desired a marker in hemodialysis patients that is easily obtained and would correlate with the clinician’s assessment of volume overload so as to raise the nephrologist’s concern. We describe the IDCR mathematically-derived calculation and tested it in three cohorts to confirm its relation to patient volume and prognosis. In outpatient hemodialysis units, IDCR can be easily calculated from post and pre-hemodialysis blood samples and reported to clinicians together with other laboratory values to alert caregivers about patients with possible volume overload. In hospitalized dialysis patients, creatinine values are often obtained daily making IDCR calculations readily available in the inpatient setting. IDCR values below 0.1 mg/dL/h should prompt caretakers to evaluate for hypervolemia and the potential need for more aggressive ultrafiltration. Very low IDCR values below 0.05 mg/dL/h should alert clinicians to an increased mortality risk. Monthly IDCR values in the outpatient setting could be an added measure for assessing and managing volume overload and mortality risk.

## Additional file

Additional file 1: Scientific basis for the Interdialytic Creatinine Rise (IDCR) as a marker of changes in body volume: derivation of IDCR as a marker of volume from the principle of mass conservation and simulation of volume gain for different IDCR values. (DOCX 537 kb)

## Abbreviations

ESRD: End-stage renal disease
HD: Hemodialysis
IDCR: Interdialytic Creatinine Rise
IDWG: Interdialytic weight gain
NPV: Negative predictive value
obs: Observations
PPV: Positive predictive value

## References

[1] Hung SC, Lai YS, Kuo KL, Tarng DC (2015). Volume overload and adverse outcomes in chronic kidney disease: clinical observational and animal studies. J Am Heart Assoc.

[2] Kalantar-Zadeh K, Regidor DL, Kovesdy CP, Van Wyck D, Bunnapradist S, Horwich TB, Fonarow GC (2009). Fluid retention is associated with cardiovascular mortality in patients undergoing long-term hemodialysis. Circulation.

[3] Agarwal R (2013). Volume overload in dialysis: the elephant in the room, no one can see. Am J Nephrol.

[4] Hecking M, Karaboyas A, Antlanger M, Saran R, Wizemann V, Chazot C (2013). Significance of interdialytic weight gain versus chronic volume overload: consensus opinion. Am J Nephrol.

[5] Flythe JE (2017). Turning the tide: improving fluid Management in Dialysis through technology. J Am Soc Nephrol.

[6] Zoccali C, Moissl U, Chazot C, Mallamaci F, Tripepi G, Arkossy O, Wabel P, Stuard S. Chronic fluid overload and mortality in ESRD. J Am Soc Nephrol. 2017;28(8):2491-7. 10.1681/ASN.2016121341 PMC5533242 28473637

[7] Dekker MJ, Marcelli D, Canaud BJ, Carioni P, Wang Y, Grassmann A, Konings CJ, Kotanko P, Leunissen KM, Levin NW, van der Sande FM (2017). Impact of fluid status and inflammation and their interaction on survival: a study in an international hemodialysis patient cohort. Kidney Int.

[8] Daugirdas JT (1993). Second generation logarithmic estimates of single-pool variable volume Kt/V: an analysis of error. J Am Soc Nephrol.

[9] Waikar SS, Bonventre JV (2009). Creatinine kinetics and the definition of acute kidney injury. J Am Soc Nephrol.

[10] Yashiro M, Ochiai M, Fujisawa N, Kadoya Y, Kamata T (2012). Evaluation of estimated creatinine clearance before steady state in acute kidney injury by creatinine kinetics. Clin Exp Nephrol.

[11] Desmeules S, Lévesque R, Jaussent I, Leray-Moragues H, Chalabi L, Canaud B (2004). Creatinine index and lean body mass are excellent predictors of long-term survival in haemodiafiltration patients. Nephrology Dialysis Transplantation.

[12] Mitch W, Walser M (1978). A proposed mechanism for reduced creatinine excretion in severe chronic renal failure. Nephron.

[13] Gardner MJ, Altman DG (1989). Calculating confidence intervals for proportions and their differences. Statistics with confidence.

[14] Doi K, Yuen PS, Eisner C, Hu X, Leelahavanichkul A, Schnermann J, Star RA (2009). Reduced production of creatinine limits its use as marker of kidney injury in sepsis. J Am Soc Nephrol.

