# Supplementary material for: The Interdialytic Creatinine Rise is a novel marker of volume overload and mortality risk in hemodialysis patients
Source: BMC Nephrol. 2018 Aug 16;19:202. doi: 10.1186/s12882-018-1008-0 (PMC6097344; doi:10.1186/s12882-018-1008-0)

Additional file:

Scientific basis for the Interdialytic Creatinine Rise (IDCR) as a marker of changes in body volume

The principle of creatinine mass conservation has been used in a variety of settings including Kt/V estimation in hemodialysis^10^ and more recently in creatinine kinetic modeling in AKI.^11,12^ The main difference in our treatment is that volume in the differential equation describing conservation of mass of creatinine is herein allowed to vary, which allows modeling of interdialytic volume gain (*IDVG*). Assuming a single compartment model for creatinine distribution and negligible renal function, the conservation of creatinine equation becomes

$d(C\cdot V)/dt =C\cdot dV/dt + V\cdot dC/dt =G-D\cdot C,$ (Eq. 1)

where *G* is the creatinine production/generation rate in mg/h, *D* is the extrarenal creatinine clearance in dL/h, *C* is the serum creatinine concentration in mg/dL, *V* is the total body water in dL, and *t* is time in hours starting after dialysis. For simplicity, creatinine change is assumed to change linearly with time:

$C=Co+IDCR\cdot t,$ (Eq. 2)

where *Co* is the initial serum creatinine value right after dialysis, and *IDCR* is the interdialytic Creatinine Rise in mg/(dL*h). Combining Eqs. (1) and (2) we get a linear ordinary differential equation:

$(Co+IDCR\cdot t)\cdot dV/dt+V\cdot IDCR=G-D\cdot(Co+IDCR\cdot t),$ (Eq. 3)

whose solution is

*V (Co, IDCR ,t )=* $\frac{Vo\cdot Co + (G-D\cdot Co)\cdot t - IDCR\cdot D\cdot t^{2}/2}{Co+IDCR\cdot t}$, (Eq. 4)

where *Vo* = *V*(t=0) or the volume right after dialysis.

A quick unit check for Eq. 4: The numerator is in mg for all three contributing terms and the denominator is in mg/dL so that the ratio of numerator to denominator is volume in dL.

Numerator:

$Vo\cdot Co$ = dL * mg/dL = mg,

$(G-D\cdot Co)\cdot t$ = (mg/h - dL/h * mg/dL) * h = (mg/h - mg/h) * h = mg,

$IDCR\cdot D\cdot t^{2}$= mg/(dL*h) * dL/h * h^2^ = mg/(h^2^) * h^2^ = mg.

Denominator:

$Co+IDCR\cdot t$ = mg/dL + mg/(dL*h) * h = mg/dL.

Volume gain is calculated as *VG* (*t*) = *V* (*Co, IDCR, t*) *- Vo,* where *Vo* is chosen to be the ideal total body water or 60% of the body weight.

The interdialytic volume gain is the volume gain after 48h of preceding dialysis:

*IDVG* (*Co, IDCR*) = *VG* (*Co, IDCR, t = 48h*).

For the purposes of simulation we used:

The creatinine generation^13^ *G* (mg/h) = 19.5 * body weight (kg) / 24,

the extrarenal creatinine clearance^14^ *D* (dL/h) *=* 0.4 x LBW (kg) / 24,

and the sample body weight of 80 kg.

All plots are completed in Matlab (The Mathworks, Natick, MA) while differential equation was solved with the help of Wolfram|Alpha by Wolfram Research.

Results for theoretical volume gain versus time for various values of *Co* and *IDCR* are presented in Fig. S1. Volume gain increases with time and negatively depends on both *Co* and *IDCR*, increasing with decreasing *Co* and also with decreasing *IDCR*. Of the two, *IDCR* is the stronger predictor as it is contained in the quadratic time term in the numerator of Eq. 4, while the initial serum creatinine concentration *Co* is a weaker predictor as it is contained in the linear time term. The plots of *IDVG* vs *IDCR* and *Co* are presented in Figures S2 and S3. The negative influence of two theoretical predictors *IDCR* and *Co* on volume is evident, with the *IDCR* being the stronger predictor for the reasons just described. Based on this simulation, *IDCR* value of less than 0.10 mg/dL/hr seems to be particularly indicative of larger than typical volume gains.

Figure S1: Volume gain (*VG*) versus time for different values of *IDCR* (mg/dL/h) and *Co* (mg/dL).


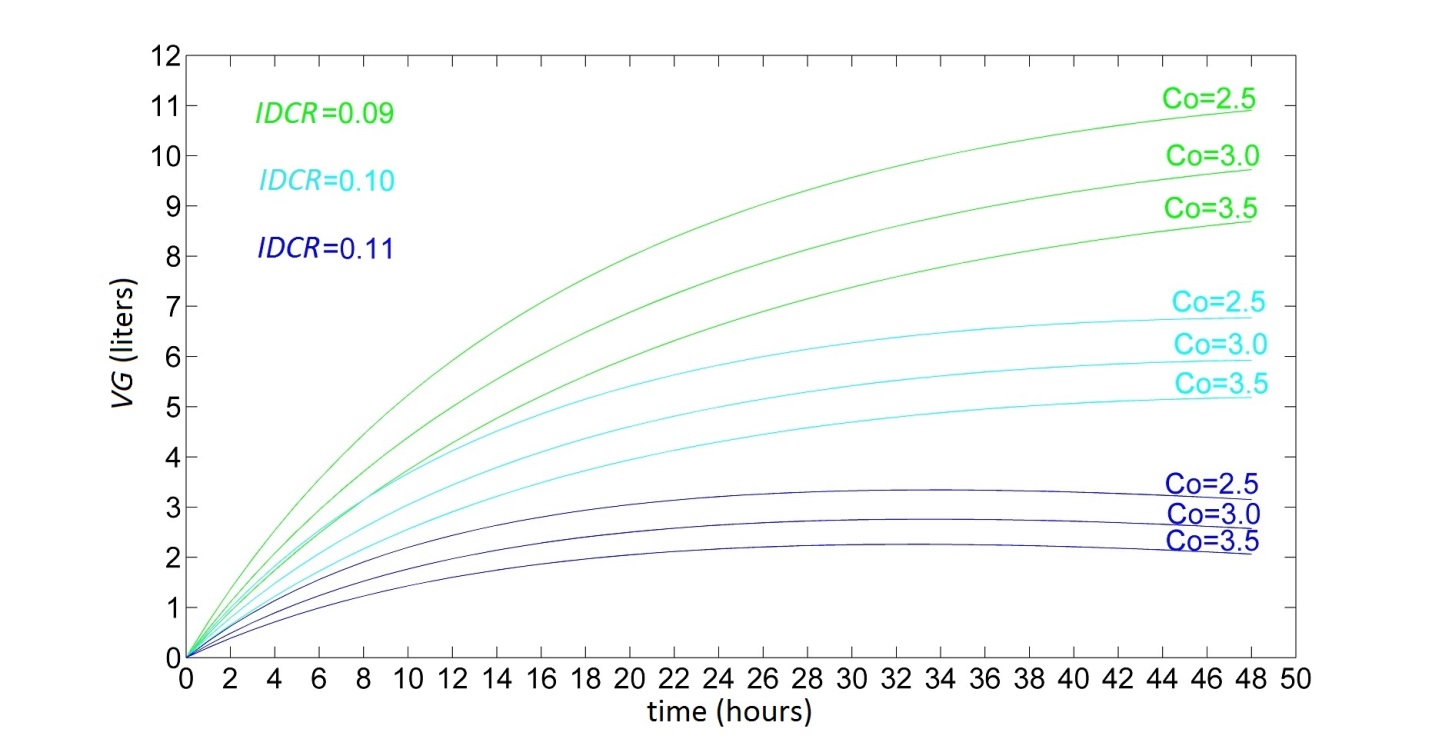


Figure S2: Interdialytic volume gain (*IDVG)* versus *IDCR* and *Co.*


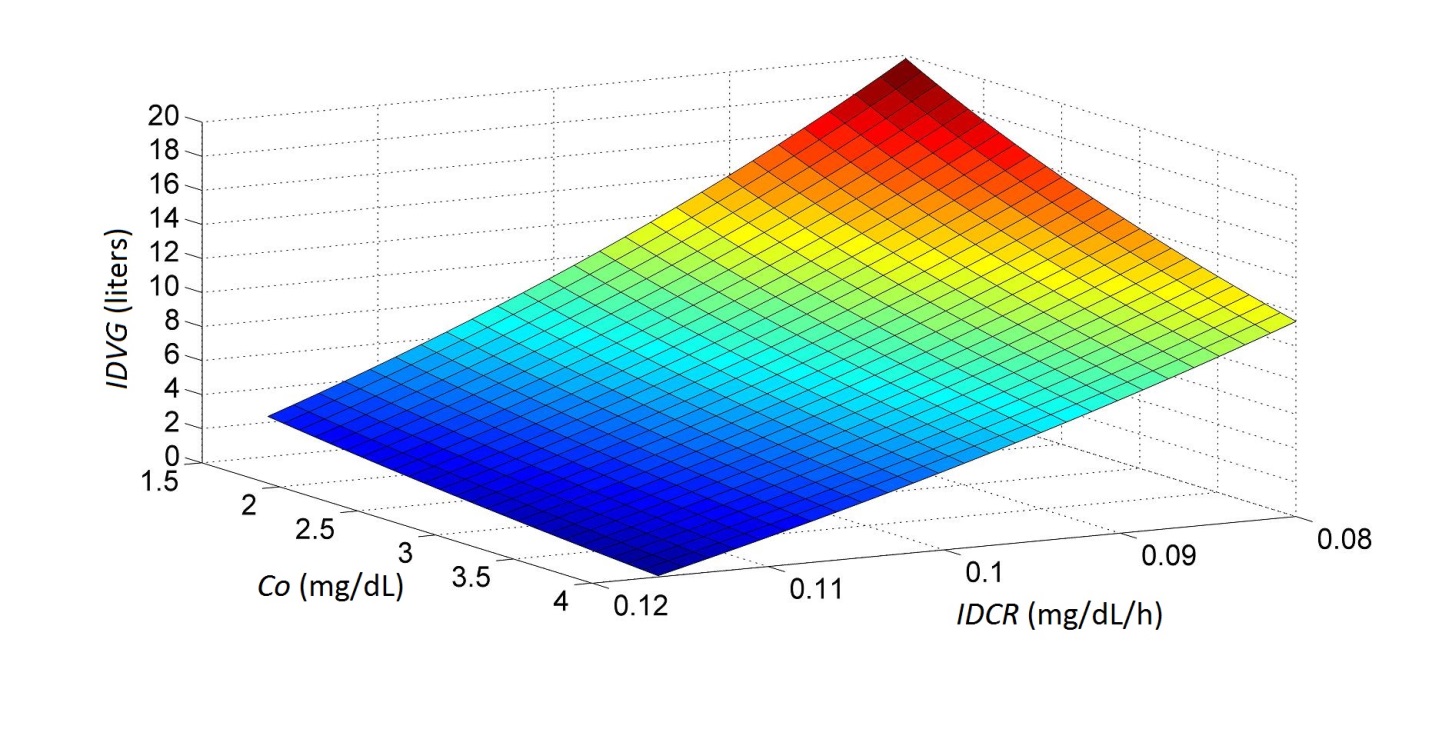


Figure S3: Contour plot of interdialytic volume gain (*IDVG)* versus *IDCR* and *Co.*


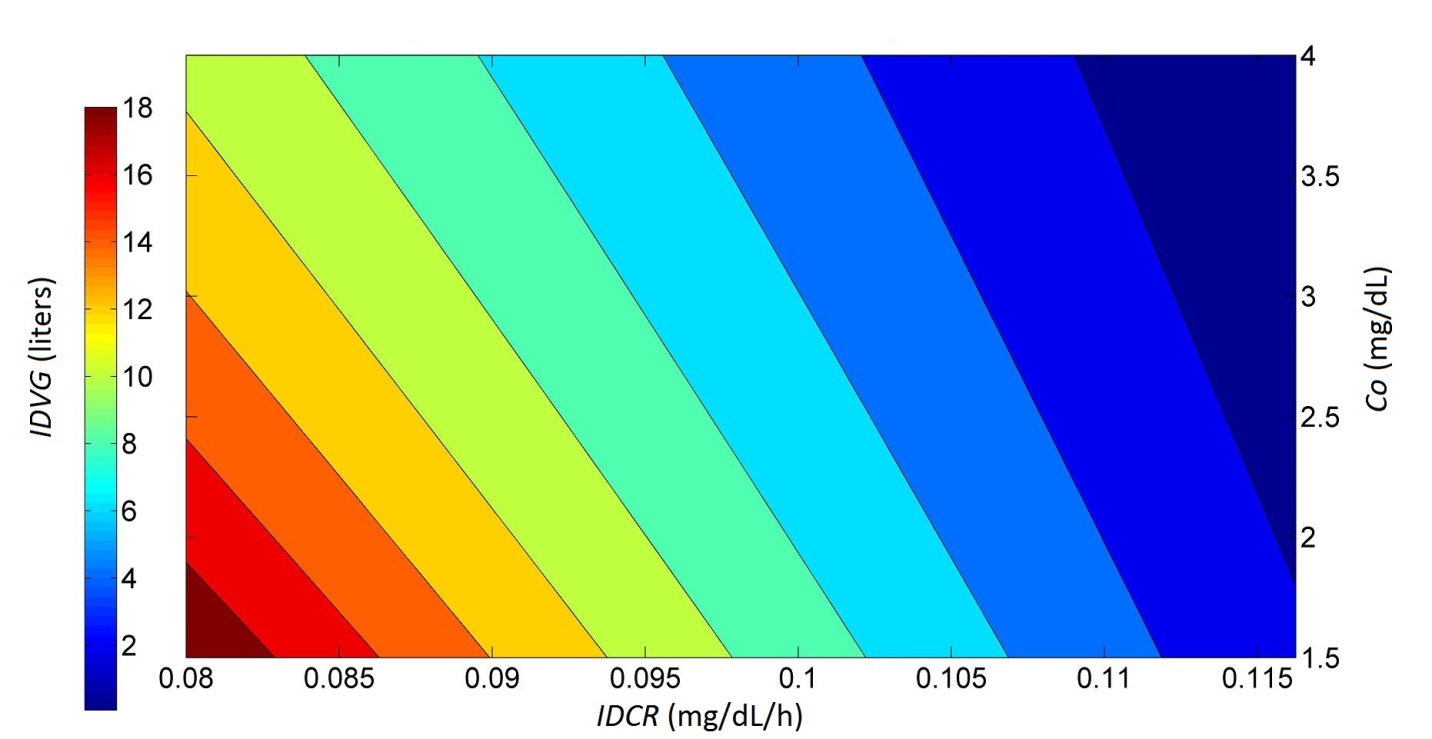

Supplement: Supplementary file 1 — Scientific basis for the Interdialytic Creatinine Rise (IDCR) as a marker of changes in body volume: derivation of IDCR as a marker of volume from the principle of mass conservation and simulation of volume gain for different IDCR values. (DOCX 537 kb) [file 12882_2018_1008_MOESM1_ESM.docx]
